# Seven reasons why binary diagnostic categories should be replaced with empirically sounder and less stigmatizing dimensions

**DOI:** 10.1002/jcv2.12108

**Published:** 2022-10-09

**Authors:** Benjamin B. Lahey, Henning Tiemeier, Robert F. Krueger

**Affiliations:** ^1^ Department of Public Health Sciences (MC 2000) University of Chicago Chicago Illinois USA; ^2^ Harvard T. H. Chan School of Public Health and Erasmus University Medical Center Rotterdam Boston Massachusetts USA; ^3^ University of Minnesota, Twin Cities Minneapolis Minnesota USA

**Keywords:** dimensional approach, stigma, taxonomy

## Abstract

**Background:**

An ongoing positive revolution advocates a new approach to the individual differences in human emotions, cognitions, and behavior that cause distress and impair functioning. This revolution endorses the long‐proposed, but still unrealized rejection of the medical model, which attributes psychological problems to a sick brain or mind. In addition, it advocates replacing the binary diagnoses used in ICD and DSM, which assume a clear discontinuity between “normal” and “abnormal” functioning, with continuous dimensions of psychological problems.

**Method:**

Selective literature review.

**Results and Discussion:**

Seven strong reasons are provided for adopting a dimensional approach.

## Graphical abstract

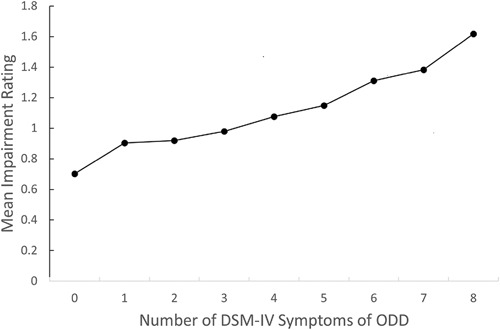

Seven logical and empirical reasons are provided for replacing binary diagnostic categories with more valid and less stigmatizing dimensions of psychological problems.

**Key points**

- Taxometric and other methods indicate that psychological problems are empirically dimensional.
- The measurement of psychological problems in dimensional terms is far more reliable and valid than categorical classification of problems.
- Categorical diagnoses ignore the unique needs of the individual.
- Categorical diagnoses encourage the reification of psychological problems and promote viewing them as unchanging rather than dynamic.
- Categorical diagnoses promote stigmatizing views of persons with problems as being fundamentally different from others.

Many psychologists and psychiatrists (Kotov et al., 2022; Lahey, 2021) believe that a tipping point has been reached in an ongoing *revolution* that advocates a new understanding of the individual differences in human emotions, cognitions, and behavior that cause distress and impair functioning across the life span. This revolution involves two key changes: First, it endorses the long‐proposed, but incompletely enacted, abandonment of the medical model, which attributes psychological problems to a sick brain or mind (Bandura, 1969). We use the term, *psychological problems,* in this paper with the same denotative meaning as *psychopathology,* but we explicitly reject terms like psychopathology, mental disorder, and mental illness because their connotative meanings cause stigma by implying that the person is no longer whole, but has a has sick mind (Lahey, 2021). We do not mean that psychological problems are the purview of only psychology rather than other disciplines. Furthermore, we certainly do not support a Cartesian mind‐body dichotomy that implies that advances in neuroscience and genetics do not help us understand psychological problems (Lahey, 2021). Individual differences in behavior are *always* accompanied by individual differences in brain and related systems, but it is unnecessarily stigmatizing to view such differences as illness.

Second, this revolution advocates replacing the binary diagnoses used in ICD and DSM^1^ with *continuous dimensions of psychological problems*. Diagnoses assume that there is a clear *discontinuity* in which a person is either “abnormal” (i.e., meets criteria for a diagnosis) or is “normal” (i.e., does not meet criteria for a diagnosis). There are no shades of gray in ICD and DSM diagnoses, even though there are nothing but shades of gray in reality. In sharp contrast, the assertion of dimensionality avers that there is continuous variation in the frequency and severity of problems—and the distress and functional impairment associated with them—across the full range of each dimension and that there is no natural or meaningful binary threshold between “having” or “not having” a psychological problem.

Child and adolescent psychologists and psychiatrists have used dimensional measures for many years (Achenbach et al., 1989; Quay, 1986). Thus, many in the field are already comfortable with dimensional assessments of psychological problems. Even if moving from categorical to dimensional assessments of psychological problems feels like a major paradigm shift to some, however, it is a necessary revolution that will be well‐worth the effort.

## SEVEN REASONS FOR ADOPTING A DIMENSIONAL APPROACH

The most compelling reasons for shifting from categories to dimensions of psychological problems are:

### Psychological problems are empirically dimensional

The categorical versus dimensional status of psychological problems has been the focus of numerous investigations using sophisticated statistical modeling to compare the fit of categorical and dimensional models to data. This literature overwhelmingly supports dimensional models of psychological problems. Haslam and colleagues presented the results of an extraordinary meta‐analysis of 183 articles using taxometric methods (methods designed to identify a category, should a category exist in data), and found consistent support for dimensional structures (Haslam et al., 2020). Latent variable modeling approaches to this issue also reach the same conclusions (Krueger et al., 2018). Psychological problems are empirically dimensional, and adoption of categorical approaches runs counter to an extensive literature supporting dimensional approaches via direct comparison with categorical approaches.

In addition, findings from genomic‐wide association studies have firmly established that all psychological problems studied to date are polygenic (Smoller et al., 2019), which means that they are influenced by the net presence or absence of large numbers of genetic polymorphisms, which each account for a very small amount of genetic variance in the phenotype. This is important because R. A. Fisher demonstrated mathematically that under plausible assumptions even modest polygenicity results in a normally distributed continuum of genetic risk (Fisher, 1918). This implies that every person has a value somewhere—from very low to very high—on every continuum of genetic liability for every kind of psychological problem. When manifestations of such genetic liability transact with the environment, the result is some level of manifest problems on the continuous phenotypic dimensions (Plomin et al., 2009).

### Dimensions are more *reliable* than binary categories

The assessment of psychological problems requires *measuring* human behavior. To best serve people seeking help, therefore, we must use the most reliable measures. Reliable measures are ones that appraise people similarly each time they are assessed within a short time frame by the same or a different assessor. Reliability of measurement is not an abstruse issue; rather, it affects everyday efforts to help persons whose behavior is causing them misery and harming their lives.

**FIGURE 1:**
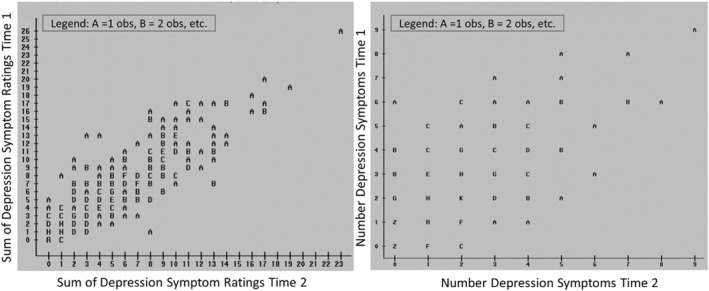
Illustration of the test‐retest reliability of parent ratings on a 0–3 scale of the sum of the 9 DSM‐IV symptoms of major depressive episode in 288 6–17 year old children and adolescents on two occasions 7–14 days apart in the population‐based Georgia Health and Behavior Study (left), and expressed at the sum of binary “symptoms” (right) (Lahey et al., 2004)

Both categorical and dimensional approaches to measurement must deal with an inherent lack of perfect consistency in what people say about their own psychological problems or those of their children, but they do so in different ways. Consider an evaluation of the test‐retest reliability of parent reports of the DSM‐IV symptoms of depression in 288 children in a larger study of psychological problems in the general population (Lahey et al., 2004). Parents rated the nine symptoms of depression on a scale of 0–3 on two occasions, 7–14 days apart. The left‐hand side of Figure 1 shows the association between the sum of these 4‐point ratings on the two occasions. Children rated lower/higher on depression at time 1 tended to be rated lower/higher at time 2, indicating imperfect, but substantial consistency in the ratings (intraclass correlation; ICC = 0.83). When the ratings were rescored as binary “symptoms” as in DSM‐IV (ratings of 2 or 3 = symptom), the ICC dropped to 0.74. Furthermore, when the symptoms were used to calculate a dichotomous “diagnosis” of major depression according to DSM‐IV criteria, Cohen's kappa was 0.44. This kappa reflected high consistency in not meeting criteria for major depression at the two time points (98.8%), but only 34.6% of children who met criteria for depression the first assessment still met criteria 7–14 days later. The magnitudes of ICC and kappa are not directly comparable, but the kappa for the categorical diagnosis was just above the conventional threshold for “fair” agreement of 0.40 (Koch et al., 1977), whereas an ICC of 0.83 is well above the conventional threshold for “excellent” reliability of 0.75 (Fleiss, 1986).

In the DSM5 field trials, *40% of the DSM5 diagnoses examined in adults did not reach the conventional cut‐off for acceptable agreement between clinicians* (Regier et al., 2013). Of great concern, the kappas for reliability of the common problems of major depressive disorder and generalized anxiety disorder were in the “unacceptable” range. In the earlier DSM‐IV field trials for children and adolescents, the reliability of externalizing diagnoses was in the barely acceptable range for parent reports of symptoms in their 9–17 year old children, but were unacceptable for youth reports of their own symptoms (Schwab‐Stone et al., 1996).

Fortunately, we can and should do better in assessing and conceptualizing psychological problems. Measuring psychological problems as continua is inherently more reliable than placing persons into binary categories.^1^ When using categorical measurement, a change to a single diagnostic criterion could change the diagnosis from ‘mentally ill’ to ‘normal.’ When measuring psychological problems dimensionally, the same amount of change does not radically change the appraisal of the person's problems.

### Dimensions are more *valid* predictors of adverse outcomes than categorical diagnoses

The greater validity of dimensional measurement is partly the result of its greater reliability, but also because continuous dimensions capture variations above and below the “diagnostic threshold” that are related to distress and impairment. Consider how diagnostic thresholds are chosen for ICD and DSM mental categories. Often this is done based solely on tradition and expert opinion, but even when data is used to choose thresholds, the process is not what most of us assume. For example, the symptoms and thresholds for DSM‐IV disruptive behavior disorders were selected using data from the DSM‐IV field trials (Lahey, Applegate, Barkley, et al., 1994; Lahey, Applegate, McBurnett, et al., 1994). Plots of numbers of symptoms against continuous measures of impairment were used to select thresholds. It is not widely appreciated that those plots almost always showed linear associations although this was stated in the reports at the time (Lahey, Applegate, Barkley, et al., 1994).

**FIGURE 2:**
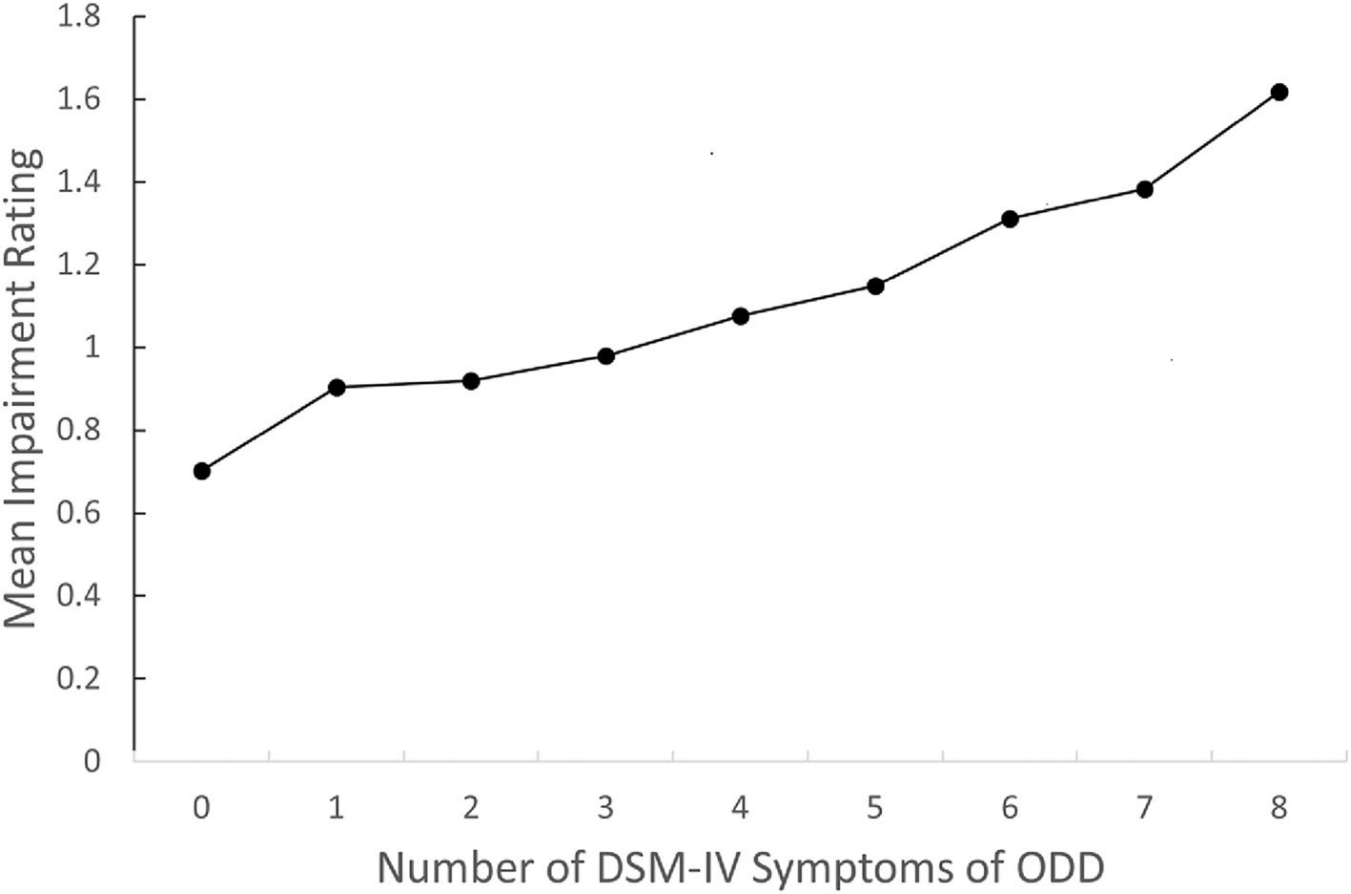
Plot of the number of “symptoms” of parent‐rated oppositional defiant disorder against means levels of parent‐rated global impairment and distress in the population‐based Georgia Health and Behavior Study (Lahey et al., 2004)

Consider the plot of the number of parent‐reported DSM‐IV symptoms of oppositional defiant disorder (ODD) against a measure of global distress and impairment in Figure 2, which is based on the Georgia Health and Behavior Study (GHBS) of a general population sample (Lahey et al., 2004). The plot shows the same linear association seen in the field trials. How can one select a meaningful diagnostic threshold based on such data? In the case of DSM‐IV ODD, an arbitrary threshold was imposed on the continuous measure of impairment to help select an arbitrary diagnostic threshold for the dimension of ODD problems (Lahey, Applegate, Barkley, et al., 1994). This was not done as an exercise in smoke and mirrors, but in a well‐meaning effort to consider empirical data when making an inherently arbitrary decision. Nonetheless, the first‐author's (BBL) participation in this effort was a signal event in his rejection of dichotomous diagnostic categories (Lahey, 2021).

Beyond the arbitrary nature of diagnostic thresholds, Figure 2 has important practical implications. Many children just below the diagnostic threshold for ODD and longitudinal studies of children, adolescents, and adults have reported that persons whose problems fall just below the diagnostic criteria for a diagnosis (“subthreshold”) are often substantially distressed and impaired and are at increased risk for meeting full diagnostic threshold for the disorder in the future (Angst et al., 1997; Balazs et al., 2013; Balazs & Kereszteny, 2014; Fergusson et al., 2005; van Os, 2014). Thus, a dimensional approach is more inclusive and facilitates the selection of graded interventions appropriate to the severity of the individual's problems (Lahey, 2021).

### Binary diagnoses are Procrustean beds that ignore the needs of the individual

Diagnostic categories are *Procrustean beds* that distort or ignore many of the specific and unique characteristic of each individual by implying that everyone who meets criteria for a diagnosis is essentially alike. This term comes to us from the Greek myth of the robber baron, Procrustes. Procrustes lived near a well‐travelled road to an important religious site. He offered lodging to wealthy travelers, but while they slept in his iron bed, he made each traveler fit the bed *exactly* by stretching some parts of their bodies and cutting off protruding parts with a sword. Diagnostic categories can act like Procrustean beds by encouraging professionals to make the problems of each individual person fit the diagnostic category by stretching some facts and ignoring others.

People very often experience psychological problems from more than one diagnostic category at the same time (Caspi & Moffitt, 2018; Lahey, 2021; Lahey et al., 2017). Focusing on a differential diagnosis distracts attention from the individual's unique combination of problems from across dimensions, each of which may be a legitimate target for intervention. This occurs because of a fundamental fact ignored by ICD and DSM: Every dimension of psychological problems is positively correlated with every other dimension. The problems that define each dimension of psychological problems do not come from *separate silos* as implied by categorical diagnostic systems. Owing to the ubiquitous positive correlations among problems, people do not exhibit the ‘symptoms’ of a single ‘mental disorder;’ they experience an *admixture of psychological problems*.

Almost no child exhibits, for example, six inattention problems and no other problems. Rather they typically also exhibit multiple other correlated psychological problems, often from more than one other dimension. Such admixtures of problems are *expected* in the dimensional approach. In contrast, in a categorical approach, admixtures of psychological problems are viewed as *violations* of the sharp boundaries that should divide supposedly distinct diagnostic categories. This encourages Procrustean distortions of the individual's problems to fit the binary diagnoses. This is a primary shortcoming of the diagnostic approach and a sufficient reason by itself for leaving it behind. Nature is complex and children and adolescents with psychological problems do not conveniently present with problems that match neatly one and only one diagnostic category description. In nature, psychological problems are dimensional, correlated, and admixed.

### Diagnostic categories encourage us to reify psychological problems

Binary categories of anything, including problematic behaviors, emotions, and cognitions, encourage us to reify the category as a set of *things* (Hyman, 2010). Psychological problems are not things; they are *individual differences* in emotions, motivations, actions, perceptions, and thinking that cause distress and impairment that are properly described by modifiers—adjectives and adverbs that refer to variations in our behavior. Dimensions promote thinking in terms of quantitative modifiers (e.g., slightly, moderately, or extremely anxious when speaking in public).

### Categorical diagnoses foster a static understanding of psychological problems

Diagnoses incorrectly imply that the individual has a relatively condition that is unlikely to change. Instead, longitudinal studies reveal that people of all ages frequently change from one categorical diagnosis to another over time (Lahey et al., 2014; Shevlin et al., 2017). The assumptions underlying systems of correlated dimensions are not inconsistent with change over time.

### Dimensional approaches promote less stigmatizing views of psychological problems

Nearly all cultures stigmatize people whose behavior causes them distress and interferes with their lives. Such stigma magnifies the challenge of having psychological problems immensely (Hinshaw, 2006, 2017). Stigma hurts us in three major ways. First, if we are embarrassed that we feel depressed, for example, that embarrassment can make us even more depressed. Second, stigmatizing psychological problems can make it more difficult for parents to seek help for their children when they might would benefit from it. The same is true for parents who often have psychological problems that interfere with helping their troubled children (Chronis et al., 2003; Chronis‐Tuscano et al., 2013). Third, the stigma felt by other people about our psychological problems can lead them to treat us as less than fully human, avoid being with us, and create barriers to employment and housing that make our lives far worse. Indeed, stigmatized and uninformed views of psychological problems often lead to unnecessary incarceration and deadly confrontations with police.

There are two separable ways in which the use of diagnostic categories in ICD and DSM promotes stigma. First, even if diagnostic categories were not currently tied to the medical model, the mere use of binary categories promotes stigma by suggesting that the individual has a problem that is qualitatively different from problems of other persons. In contrast, dimensional portrayals of psychological problems reveal the quantitative variation in dimensions of problems from very low to high in the population. It should be easier to reduce the stigmatization of problems that are simply viewed as extreme on a natural continuum enough to warrant intervention than for problems that are viewed as fundamentally different in kind.

Second, the fact that binary categories are tied to the medical model in ICD and DSM makes the problem of stigma much worse. The view that psychological problems are the result of biological problems dates back at least 2400 years to Hippocrates, who believed that psychological problems were manifestations of imbalances in the fluids of the body. For centuries, this model competed with views that psychological problems were caused by gods, demons, or moral turpitude, but Hippocratic thinking became the dominant view in the Western world in the 1800s in the guise of the *medical model* of psychological problems. This happened because of the truly astonishing discovery by Richard Krafft‐Ebing and others that the bacteria that causes syphilis often infects the brain resulting in the debilitating syndrome of psychosis and dementia known as general paresis. When the successful treatment of syphilis with penicillin was perfected a hundred years later during World War II, the previously high number of persons with incidence cases of general paresis fell to nearly zero in Western countries. It was an electrifying scientific triumph! Understandably, this advance in alleviating human suffering led to the optimistic belief that every kind of psychological problem would eventually be found to be caused by germs affecting the brain. This fostered the belief that psychological problems are actually medical problems and that medical doctors are the professionals who should treat psychological problems.

There is, of course, every reason to provide medical treatments to persons with treatable infections that cause psychological problems. Very few other infections that cause psychological problems subsequently have been discovered, however. This fact should have led to a delimited medical model of psychological problems, but it did not. Very unfortunately, the medical model took on a much broader *metaphorical meaning* when few additional links between germs and psychological problems were discovered. The logic of the modern medical model was extended to metaphorical “diseases of the mind”—syndromes of mental ‘symptoms’ without known biological illnesses (Klerman, 1977).

Most psychologists and psychiatrists active today were trained to believe that they can discern the difference between ‘normal’ and ‘abnormal’ minds, and thereby ‘diagnose mental illnesses.’ Our view is that this is an entirely fictional and baseless notion that is toxic to people. Telling people that psychological problems are the result of terrifying illnesses of the mind promotes the worst forms of stigma.

Over 50 years ago, psychologist Albert Bandura (1969) pragmatically defined psychological problems without reference to biological illness simply as “…behavior that is harmful to the individual or departs widely from accepted social and ethical norms…” (p. 10). Psychiatrist Thomas Szasz similarly advocated replacing medical model terms such as mental illness with the less judgmental phrase, *problems in living* (Szasz, 1960, 1974). Szasz has been widely misunderstood as denying the existence psychological problems. He explicitly did not do so, but he denied the meaningfulness of the concept of mental illnesses based on an analogy to medical illness.

Nonetheless, we stigmatize psychological problems partly through the words we use, often with the best of intentions. Most of us refer to psychological problems with medical model terms such as *mental illnesses, mental disorders, psychopathology*, or *mental health problems.* We often use these medical model terms in caring ways to imply that the psychological problem is not the person's fault, but is the result of their mental illness. These are profoundly stigmatizing terms, however. They say that your psychological problems are the result of your *illness, disorder, and pathology*—that you have psychological problems because your mind is *sick!* How can that not worsen stigma?

## “ORDINARINESS” OF PSYCHOLOGICAL PROBLEMS

To fully fight stigma, we need to recognize that psychological problems are *ordinary.* This emphatically does not mean that they are unimportant and can be ignored. Psychological problems often make people miserable and interfere significantly with their lives, sometimes in ways that are nothing short of tragic. Nonetheless, psychological problems are *ordinary* in two very important ways: First, psychological problems are not the product of diseased minds or brains, *they arise through the same normal biological and psychological processes as any other aspect of behavior* (Lahey, 2021). That does not mean that there are not some forms of problem behavior that are distinctly different from typical behavior, such as hallucination and delusions. It is simply to assert that even extreme forms of psychological problems arise from the same processes as all behavior (Lahey, 2021). Second, recent studies have revealed that psychological problems are so much more *common* in the population than we realized that they cannot be considered to be anything but ordinary (Moffitt et al., 2010).

When we recognize that the *great majority of us* will experience problems like fear, anxiety, sadness, or cravings for deadly substances at some time in our lives, it will be harder to stigmatize psychological problems. Psychological problems are not rarefied things experienced by a few people with diseased minds; they are quite ordinary things experienced by nearly all of us. Several large longitudinal studies of the general population in several countries have been conducted in which the same individuals were persons were asked about their psychological problems multiple times from early adolescence through middle adulthood. These studies (Schaefer et al., 2017) tell us that an eye‐popping *80% of people in the general population* met DSM diagnostic criteria for at least one mental disorder at least once during the decades they were studied. The level of diminished functioning was not great in all cases, but psychological problems are always a burden. Note, too, that these studies only reported the prevalence of meeting *full DSM diagnostic criteria* for a mental disorder. Far more people reported psychological problems that were just below the ‘official’ DSM threshold for a diagnosis. Therefore, even the remarkable finding that *most* of us will meet full DSM criteria for a mental disorder at some point in our lives understates the ordinariness of psychological problems.

## IMPLICATIONS OF A DIMENSIONAL APPROACH FOR SERVICES

If psychological problems are viewed in dimensional terms, where on the continuum is intervention useful and appropriate? How would mental health and school systems decide who is eligible for scarce professional services? There are no natural thresholds between adaptive behavior and psychological problems. Rather, all evidence to date indicates that as problems gradually increase across continua, so do distress and functional impairment (Lahey, 2021; Lahey, Applegate, Barkley, et al., 1994; Lahey, Applegate, McBurnett, et al., 1994; Lahey et al., 2008).

It has been argued that categorical thinking is justified given the need in medical practice to make treatment decisions that are inherently dichotomous. Indeed, administrative and reimbursement requirements impede the movement towards a continuum approach. To address this issue, some have advocated developing a triage and service delivery based on severity, functional difficulties, and prognosis to direct limited resources to those most in need of treatment (McLennan, 2016). Diagnoses arguably provide false comfort in making dichotomous treatment decisions partly because this approach overestimates the accuracy of the link between diagnosis and treatment.

If we do not use diagnostic thresholds, how do we make the *inherently binary decision to treat or not treat* (Widiger, 2019)? One pragmatic answer is that persons with problems or the adult caregivers of minor children could legitimately decide, in consultation with teachers and professions that individual's thoughts, feelings, and actions are distressing or interfering enough to seek help *at any point* on the continuum. Specifically, at any point on the continuum where the distress and impaired functioning that the individual currently experiences—and may experience in the future if help is not provided—is judged to outweigh the usually small risks inherent in receiving help, then help is justified. No one has to decide that the individual has a mental illness to receive help.

This approach is subjective to be sure. Psychological problems and distress and functional impairment can be reliably measured across the life span, but decisions on the points on these continua where the expected benefits outweigh the likely costs cannot currently be based on sound normative data. Although that is true, the alternative is keep the current system, which is based on binary diagnoses measured with unacceptable reliability and validity.

It is easy to imagine a system that provides help to all those who would benefit from it without requiring them to have a diagnosis of a mental illness. This might be politically difficult to achieve, but it would be just and it would not be impractical. Adopting such a non‐stigmatizing dimensional would require a revamping of policies and funding strategies for treatment, however, and it would require controlled trials to evaluate the clinical utility of approaches to providing services based on dimensional assessments versus the current categorical approaches. A defensible dimensional approach to decision making would require standardized continuous measurement of psychological problems and functional deficiencies. This could improve cost‐effectiveness of treatment allocation and would almost certainly reduce structural inequalities. Currently, those most need of services often have the greatest barriers to obtaining them (Kazdin, 2019; Velasco et al., 2020).

The obstacles to such changes would likely be enormous. In countries like the United States, insurance for services of psychological problems is provided by health insurance companies, who almost certainly would deny payment for services for psychological problems if they are no longer considered to be “health” problems. Services from a national health service may be similarly affected. Schools should be a position to change to a dimensional approach more easily, but the legislation that authorizes services for children with psychological problems would need to be considered carefully and potentially revised.

Would it be affordable for people and families to be allowed to decide freely for themselves when they need professional help? Services for psychological problems cost money. Currently, psychologists, special educators, psychiatrists, and other physicians are the gatekeepers to such services. Only persons given a reimbursable DSM or ICD diagnosis of a mental disorder can receive services for psychological problems without paying directly for it themselves in nearly every country. Whether you live in the United States where private and government health insurance plans pay for psychological services—if you are lucky enough to have good health insurance—or live in one of the many countries in which taxpayer‐supported services for psychological problems are provided essentially for free, you cannot receive those services without a qualifying diagnosis in virtually every case. Your diagnosis is your only ticket to services, unless you are willing to pay for them yourselves. Government systems and insurance companies likely believe that this is necessary control the costs of services.

Nonetheless, societies could decide to provide services for psychological problems to all who seek them for one of three reasons. First, it may not actually increase the number of people who receive services very much. As hard as we fight stigma, many families and individuals are still reluctant to seek services because of stigma and other barriers. Indeed, the number of people seeking professional services may not increase very much if they are free to do so without a diagnosis, particularly in the beginning. Thus, the increase in cost may not be great; we will not know unless we try.

Second, some societies may decide that providing services to all who feel that they need them would actually save the society money. Psychological problems are extremely costly to society in terms of reduced economic productivity and increased physical health problems. There is every reason to believe that increased expenditures for evidence‐based, cost‐effective methods of preventing and reducing psychological problems would be more than repaid by reductions in the large economic costs of psychological problems to society (Cuijpers et al., 2021; Kazdin, 2019; Moffitt, 2019).

Third, even if it resulted in increased costs, it would not be unreasonable for a society to decide that spending tax money on reducing psychological problems in everyone who felt the need for it would be one of the most justifiable ways in which public monies could be spent. It may make sense from the perspective of health insurance companies and government health systems only to reimburse services that treat “medical conditions,” but this economic‐based practice forces psychiatrists, educators, psychologists, and other helping professionals to address psychological problems in medical terms. This leads all of us to think about psychological problems in genuinely harmful ways, usually without realizing that we are doing so.

## AUTHOR CONTRIBUTIONS

**Benjamin B. Lahey**: Conceptualization, Formal analysis, Writing – original draft, Writing – review & editing. **Henning Tiemeier**: Conceptualization, Writing – original draft, Writing – review & editing. **Robert F. Krueger**: Conceptualization, Writing – original draft, Writing – review & editing.

## CONFLICTS OF INTEREST

Benjamin B. Lahey and Henning Tiemeier both serve on the JCPP *Advances* Editorial Advisory Board. Robert F. Krueger declares that they have no competing or potential conflicts of interest.

## ETHICAL CONSIDERATIONS

No new human participant data were created or analyzed in this study.

## Data Availability

Data sharing is not applicable to this article.

## ENDNOTE

^1^
The various editions of the International Classification of Diseases published by the World Health Organization and the Diagnostic and Statistical Manual of Mental Disorders published by the American Psychiatric Association.
